# Relational care in palliative care units: a qualitative study of low-threshold volunteer hospice conversations

**DOI:** 10.1186/s12913-026-14399-0

**Published:** 2026-04-11

**Authors:** Carolina Müller, Daniel Authier, Christine Steger-Arand, Roland Braun, Wolfgang Herr, Annette Schnell, Michael Rechenmacher

**Affiliations:** 1 Clinic and Polyclinic for Internal Medicine III, University Hospital Regensburg, Regensburg, Germany; 2 Center for Palliative Care, University Hospital Regensburg, Regensburg, Germany; 3 Hospital Barmherzige Brüder, Clinic for Palliative Care, Regensburg, Germany

**Keywords:** Palliative care, Volunteers, Psychosocial support systems, Interpersonal relations, Ethics, Clinical

## Abstract

**Background:**

Palliative care aims to address psychosocial needs alongside medical treatment. In inpatient settings, however, organisational constraints may limit opportunities for sustained relational support. Volunteer-based conversation offers represent a potential service-level response to these gaps, yet little is known about how such low-threshold models are implemented and experienced within routine inpatient care.

**Methods:**

This qualitative descriptive interview study explored the experiences of twelve trained volunteer hospice companions involved in a low-threshold conversation offer on two palliative care units in Germany. Semi-structured individual interviews were analysed using qualitative content analysis following Kuckartz, with inductively developed categories and independent double coding.

**Results:**

Volunteers described the conversations as a distinct relational care practice grounded in presence, attentiveness and deliberate restraint rather than intervention. Care was enacted through responsiveness to patients’ momentary needs, including respect for silence, refusal and non-verbal interaction. The ward-based interdisciplinary setting and effective symptom control were perceived as key enablers of psychosocial conversations, while the rotating and low-binding format entailed specific emotional and organisational demands, underscoring the importance of supervision and role clarity.

**Conclusions:**

Low-threshold, non-prearranged volunteer conversations represent a distinct relational care practice in hospital palliative care. They may support autonomy and dignity through non-imposition and situational responsiveness, and they highlight the ethical responsibility of institutions to provide structural support, supervision and clearly defined roles for volunteers.

**Supplementary Information:**

The online version contains supplementary material available at 10.1186/s12913-026-14399-0.

## Introduction

Palliative care aims to alleviate suffering and support quality of life for people with life-limiting illness, including psychosocial and existential needs [1]. In hospital palliative care units, however, high clinical workload, time pressure and rapidly changing patient trajectories can limit opportunities for sustained presence and open-ended conversations. Patients and relatives have repeatedly reported unmet needs such as the wish to be heard, emotional support and opportunities to express fears or ambivalence, particularly in inpatient settings characterised by task-oriented care [2, 3].

Volunteer hospice companions are an established component of hospice and palliative care services. Most existing volunteer models rely on continuity-based accompaniment, with volunteers assigned to individual patients over time or engaged through pre-arranged contacts [4, 5]. By contrast, less is known about volunteer-led conversation offers that are embedded in inpatient routines, occur without prior request and are deliberately non-binding [6].

The model evaluated in this study represents such a rotating, low-threshold form of psychosocial support. In two participating units, a local hospice association organised the pool of trained volunteer hospice companions, overseeing recruitment, basic training and the programme overall. Day-to-day scheduling and on-site coordination were jointly managed by the association’s volunteer coordinator and designated clinical leads on each palliative care unit. The model is characterised by non-prearranged encounters within a fixed weekly time frame, rotating volunteer shifts, and the absence of ongoing assignment to individual patients. This open structure is intended to lower access barriers, preserve voluntariness and enable responsiveness to patients’ momentary needs within a dynamic institutional setting. To date, such rotating low-threshold conversation offers have rarely been examined empirically, particularly regarding their relational dynamics, facilitators and challenges for volunteers [7].

Conceptually, these encounters can be understood as a form of relational care that is less oriented towards specific conversational content than towards presence, recognition and responsiveness. Work on relational autonomy and care ethics suggests that such practices may support autonomy and dignity not by maximising choice, but by protecting patients from overload and subtle forms of social pressure [8, 9].

Against this background, the present study explores how volunteer hospice companions experience and enact this form of support on a hospital palliative care unit, and how the ward setting shapes these practices. Specifically, the study examines how low-threshold, non-prearranged conversations are perceived by volunteers, which relational processes they involve, and which conditions facilitate or hinder their provision.

## Methods

### Study design

This study was designed as a qualitative descriptive interview study exploring the experiences of volunteer hospice companions involved in a low-threshold conversation offer on a hospital palliative care unit. A qualitative descriptive approach was chosen to closely represent participants’ accounts without aiming to develop a formal theory [10].

The study followed an exploratory design and was reported in accordance with the Consolidated Criteria for Reporting Qualitative Research (COREQ) guideline for qualitative interviews [11].

### Setting and context

The study was conducted on two specialised inpatient palliative care units in a regional centre in South Germany: one located at a university hospital and one at a large tertiary care hospital. Both units operate within comparable organisational and structural frameworks, including identical certification standards, interdisciplinary team composition, and care processes.

Volunteers were recruited and supported by the cooperating hospice association within its structured volunteering programme. Before joining the conversation offer, all volunteers completed standardised basic hospice training combining teaching and reflective group work on communication with seriously ill people, core palliative care principles, grief and loss, and end-of-life ethics. They also participated in mandatory monthly group supervision facilitated by experienced hospice professionals. On the wards, volunteers were introduced and supported by the association’s volunteer coordinator and designated nursing contacts, who assisted with practical issues and information exchange during visits. The weekly visit time was agreed in advance with the ward teams and coordinated with routine medical and nursing activities. Volunteers did not take part in formal interdisciplinary team meetings; however, they typically exchanged brief updates with nursing staff at the beginning and end of each visit. With prior patient consent, nurses provided short pre-visit information on current status and relevant preferences or restrictions, and volunteers could share pertinent observations after the visit within the same consent framework.

As part of this cooperation, trained volunteer hospice companions regularly offered low-threshold, non-prearranged conversations to patients on both units. The offer consisted of weekly visits within a fixed time frame, a rotating pool of volunteers without permanent assignment to individual patients, voluntary participation by patients, and a focus on psychosocial support rather than predefined therapeutic goals.

Patients were informed about the offer by nursing staff as part of routine ward communication and could decide spontaneously whether to engage with volunteers at each visit. Encounters with volunteers were clearly distinguished from professional psychosocial services such as psycho-oncology and chaplaincy in terms of role, training and documentation responsibilities.

### Participants and sampling

Participants were volunteer hospice companions actively involved in the conversation offer at the time of the study. A purposive sampling strategy was applied to include all volunteers with direct experience of the intervention. Inclusion criteria were completed training as a hospice companion, active participation in the conversation offer, and willingness to participate in an interview.

Thirteen volunteers were invited via email; twelve participated. All participants were affiliated with the same hospice organisation. Given the focused research aim and inclusion of all volunteers involved in the project, the sample was considered sufficient to capture a range of relevant experiences, while transferability to other contexts remains limited.

### Data collection

Data were collected through semi-structured individual interviews, allowing participants to reflect openly on their experiences while ensuring coverage of key topics relevant to the research aim. An interview guide was developed by the research team based on the study objectives, existing literature on hospice volunteering, and preliminary team discussions.

The guide included open-ended questions addressing experiences with the conversation offer, perceived benefits for patients and relatives, facilitating and hindering factors related to the ward setting, and perceived challenges and emotional demands associated with the role (see Appendix 1).

Interviews were conducted in German by CM (doctoral researcher) between July and November 2024, either face-to-face (*n* = 6) or online (*n* = 6), in a quiet and private setting. The average interview duration was approximately 50 min (range: 27–66 min). CM had no prior relationship with participants. All interviews were audio-recorded with consent, transcribed verbatim, checked for accuracy by MR, and anonymised. Quotations were translated into English for publication with attention to preserving meaning.

### Data analysis

Data were analysed using qualitative content analysis following Kuckartz [12], employing a structuring approach with inductive category development. Analysis involved repeated reading of transcripts, inductive development of main categories based on the research questions, and iterative refinement of subcategories through comparison across interviews.

A coding framework with category definitions and anchor examples was developed iteratively and discussed in team meetings. All transcripts were coded independently by CM and MR using MAXQDA software. Initial coding was compared to establish a shared understanding of the category system; discrepancies were resolved through consensus in the research team. Analytic decisions were documented in memos to enhance transparency and dependability [11].

### Reflexivity and research team

The research team had backgrounds in palliative care, nursing science and qualitative research, including professional proximity to hospital-based palliative care. Potentially positive preconceptions about volunteering and relational care were addressed through regular reflexive discussions. After the first interviews, the interviewer and a senior researcher reviewed initial codes for assumption-driven interpretations and subsequently attended to ambivalent and disconfirming accounts during subsequent coding. Interviews were conducted by a doctoral researcher not involved in clinical care to reduce social desirability and support an open interview atmosphere.

### Ethics approval and reporting

Ethics approval details are provided in the section “Ethics approval and consent to participate.” The completed COREQ checklist is provided in Appendix 2.

## Results

Twelve volunteer hospice companions participated in the study. Participants were predominantly female (11 female, 1 male) and had more than two years of experience in hospice volunteering, with between 6 and 19 months of involvement in the weekly conversation offer. All were actively involved in the low-threshold conversation offer on the hospital palliative care units during the study period. Interviews explored how volunteers experienced and enacted this form of psychosocial support within the ward context, including perceived opportunities and challenges.

Qualitative content analysis resulted in three main categories with sixteen subcategories: (1) open-endedness enables situationally responsive relational support, (2) the ward-based setting facilitates psychosocial conversation offers and (3) openness of the offer generates specific emotional and structural demands. No additional minor themes emerged that warranted separate categories beyond this framework; rather, variations in experiences were captured within the subcategories below. For transparency, subcategories are numbered (e.g., 1.1–1.6). Quotation references indicate the interview ID and the coded transcript segment number as exported from MAXQDA (e.g., Interview_11:54). To preserve anonymity, quotation descriptors were kept minimal and reported only in aggregate. Given the small, context-specific sample from a single hospice organisation, reporting additional individual attributes would have increased re-identification risk.

## Open-endedness enables situationally responsive relational support

Across interviews, volunteers described the conversation offer less as a goal-directed intervention and more as a relational practice shaped by what patients (and sometimes relatives) needed or were able to engage with “in the moment”. This openness included the possibility that encounters would remain largely non-verbal, that topics would shift quickly, or that patients would decline contact.

### Human presence as a core element of support

Volunteers repeatedly framed “being there” as an active form of support. Presence could mean sitting quietly, offering time without pressure, or, where appropriate, gentle physical proximity. Non-verbal contact was described as meaningful particularly when patients were exhausted, distressed, or unable to talk:

Sometimes there’s no conversation at all, you can hold a hand, stroke [them]. And then you notice that maybe they become calmer. And that’s how it is, however it is, it’s always good. (Interview_09:14)

Importantly, volunteers portrayed this presence not as “doing nothing” but as deliberate availability, staying with what is, rather than trying to change it.

###  Relief through attentive listening with full presence

When patients or relatives wished to talk, volunteers perceived a frequently unmet need to be listened to without interruption, time pressure, or clinical agenda. Listening was described as a form of relief that allowed burdensome thoughts to surface, including ambivalence and emotions that are difficult to voice elsewhere:

…then they open up and say how they are: it’s hard for me, it’s a burden, I’m sad, I’m relieved if it will be over soon … finally being able to talk about themselves. (Interview_10:136)

In these accounts, the supportive element lay less in specific “advice” than in creating space, letting people speak, pause, or repeat themselves without being redirected.

### Psychosocially supportive conversations beyond family and staff

Beyond listening, volunteers also described facilitating more active psychosocial conversations, often addressing sensitive concerns that patients hesitated to share with relatives in order not to “burden” them. Volunteers interpreted their role here as providing a safe conversational outside-position: close enough to be emotionally present, but distant enough to be free of familial entanglement and clinical responsibility.

If it fits, it can be a very intense relationship… and often it’s simply that they talk with us about things they wouldn’t discuss with relatives, because they don’t want to make their hearts heavy. (Interview_09:60)

These conversations were described as intensive and often centred on worries about burdening family members, unresolved conflicts or existential questions.

### A shift in perspective and distraction from ward routine

Volunteers frequently positioned their encounters in contrast to the task-oriented rhythms of the ward. They experienced their role as offering patients a different “register” of interaction, less focused on symptoms, treatment, or organisational routines, and more on personhood and everyday meanings:

It’s another perspective … another level. (Interview_10:50)

Volunteers described bringing an “outside” perspective that was not primarily illness- or treatment-focused.

### Voluntariness and respect for refusal

A recurring feature of open-endedness was a strong emphasis on non-imposition. Volunteers described it as ethically and practically essential to notice subtle signals of reluctance and to withdraw without persuasion. Respecting refusal was not framed as a failure but as part of maintaining a low-threshold, patient-led offer:

You mustn’t impose anything on anyone. (Interview_08:16)

Volunteers described it as essential to notice and respect refusal and to end encounters whenever patients signalled that they had had enough.

### Interest in the person and individual concerns

Finally, volunteers highlighted that meaningful conversations depended on genuine interest in the individual person, not only the illness situation. They described “showing interest” and asking about personal concerns as a precondition for patients to speak about their lives or current worries:

First, show interest … really listen. (Interview_11:28)

In this sense, open-endedness was anchored in a relational stance: approaching without a script, but with attentiveness to what matters to this particular person right now.

## The ward-based setting facilitates psychosocial conversation offers

Volunteers attributed several enabling conditions directly to the inpatient palliative care context. The ward was described as shaping who could be approached, what kind of conversations were possible, and how safe volunteers felt while engaging in emotionally demanding situations.

### Non-binding encounters with unfamiliar volunteers

The rotating structure and absence of prior relationship were experienced as creating a non-binding conversational space. Volunteers believed that some patients spoke more openly precisely because volunteers were not part of the family system and not responsible for clinical decisions:

…maybe not with very close relatives, but with people who have enough distance. (Interview_11:60)

This distance was framed as relationally productive: it could enable disclosure without fear of consequences, expectations, or upsetting loved ones.

### Informational exchange with nursing staff

Before approaching patients, volunteers often received brief, situational information from nursing staff (e.g., current circumstances, family context, practical considerations). Volunteers described this as important for avoiding inappropriate timing and for approaching patients more sensitively. At the same time, they emphasised that such exchange presupposed consent and careful handling of sensitive information:

It really helps … that we have this information. (Interview_02:80)

Here, the ward context enabled a form of coordination that volunteers contrasted with home visits, where they might arrive with less situational overview.

### Sense of belonging to the ward team

Many volunteers reported feeling appreciated and welcomed on the unit, describing a partial sense of being “in the team” during their shifts. This was experienced as motivating and as supporting confidence in interacting with patients:

…you almost feel like part of the team. (Interview_05:92)

However, volunteers also implicitly positioned themselves as distinct from professional roles, suggesting that “belonging” was situational and did not erase role boundaries.

### Protected environment and good symptom control as prerequisites

Volunteers described the presence of qualified staff as providing a sense of safety. Compared with accompanying patients at home or in care homes, the ward context reduced the perceived risk of being “alone” with acute medical deterioration or highly distressing symptoms:

There I never feel alone. (Interview_04:62)

This protection was experienced as enabling volunteers to remain focused on psychosocial presence without feeling responsible for medical action.

### Good physical symptom control as a precondition for conversations

Finally, volunteers repeatedly linked the possibility of meaningful psychosocial conversations to the characteristic palliative care focus on symptom control. When patients were medically stabilised, volunteers felt that attention could shift toward emotional, existential, or relational concerns:

A great prerequisite to be able to focus on more emotional things. (Interview_02:46)

Thus, in volunteers’ accounts, specialised palliative care functioned as a structural precondition for psychosocial openness, not merely as a clinical backdrop.

## Openness of the offer generates specific emotional and structural demands

While open-endedness and the ward setting enabled psychosocial support, volunteers also described burdens that arose specifically from the low-threshold, rotating, and time-bounded character of the offer. These demands were portrayed as recurring and, to some extent, structurally built into the format rather than as individual shortcomings.

### Supervision as part of psychological self-care

All participants emphasised the importance of regular supervision for processing burdensome experiences:

Our four-weekly supervision covers a lot … that works quite well. (Interview_11:72)

This framing positioned supervision as a normal, expected element of sustainable volunteering rather than as a response to exceptional crises.

### Limited temporal flexibility and coordination with ward routines

The fixed time window and the spontaneity of approaching patients were also seen as potentially problematic. Volunteers highlighted the risk of encountering patients at inconvenient moments within a sequence of ward interruptions (staff entering, relatives visiting). One volunteer illustrated how a non-prearranged visit could feel intrusive from the patient perspective:

I don’t know if I would have wanted that. (Interview_06:28)

Rather than rejecting the model, volunteers described this as a coordination challenge that required sensitivity to timing, quick situational assessment, and clear withdrawal when appropriate.

### Emotional demands of serial first encounters with unfamiliar patients

A core burden of the rotating model was the repeated requirement to enter unknown rooms and meet unknown situations in quick succession. Volunteers described a brief but intense moment of uncertainty “at the door”, including being confronted with unexpected biographical realities:

That short moment … is always a challenge. (Interview_11:54)

This demand differed from continuity-based accompaniment, where volunteers could gradually adapt to a person’s situation over time. In the low-threshold format, emotional adjustment and relational attunement had to happen rapidly and repeatedly.

### Lack of information about subsequent patient trajectories

Several volunteers found it burdensome that the model provided little or no feedback about what happened to patients after the conversation. Some expressed a wish for follow-up or simply to know whether a patient had died or been discharged, describing not-knowing as emotionally difficult:

Otherwise I don’t know what happens to the patient. (Interview_04:38)

This absence of outcome information was not described as a minor inconvenience but as affecting volunteers’ sense of closure, especially when an encounter had felt intense or when a patient remained salient in memory.

### Confrontation with younger critically ill patients

Finally, volunteers reported that encounters with younger patients could be particularly affecting. They perceived the patient population included relatively young individuals, which intensified the emotional impact given proximity to death.

That there are so many young people … that really affected me. (Interview_03:77)

In these accounts, “young age” intensified the emotional salience of the encounter and increased the need for reflective processing and peer/supervisory support.

## Discussion

The present qualitative study shows that low-threshold, non-prearranged conversations provided by trained volunteer hospice companions on two palliative care units are perceived by volunteers as a distinct form of relational care. Central elements of this practice are open-ended presence, attentiveness to patients’ momentary needs and a deliberate stance of non-imposition, including respect for silence and refusal. The ward-based setting was experienced as both an enabler of psychosocial conversations, through interdisciplinary collaboration and effective symptom control, and as a source of specific emotional and structural demands related to the rotating, low-binding format.

The findings indicate that volunteer hospice companions primarily understand the conversation offer as a relational and open-ended form of care rather than as an intervention with predefined goals or intended outcomes (see Results, sections “Human presence as a core element of support”–“Interest in the person and individual concerns”). Core elements of this practice include presence, time and situational responsiveness to patients’ needs. Care is thus defined less by conversational content than by “being there” and a willingness to remain emotionally available. This understanding aligns with scholarly work conceptualising communication at the end of life as a relational process in which emotional presence and recognition constitute key dimensions of quality [3, 13]. Our understanding of relational presence and situational responsiveness aligns directly with recent findings. In inpatient palliative care settings, hospice volunteering is described as contributing a form of “spontaneity” that arises precisely through non-standardized, situationally intuitive acts of care, ranging from small gestures and everyday forms of closeness to fostering a “family-like” atmosphere that at times disrupts institutional routines [14]. This aligns with our categories 1.1–1.6, which depict low-threshold conversations as moment-oriented, needs-responsive practices. Concepts of compassion similarly emphasise that care becomes particularly meaningful where suffering cannot be resolved but must be accompanied [15]. Volunteer conversations are therefore often experienced as meaningful not primarily through specific topics but through relational qualities such as being heard, recognition and mutual presence, supporting an interpretation of the offer as relationship-centred rather than outcome-centred care [16].

Beyond relational qualities, the findings also point to a temporal dimension of care that warrants further consideration. From this perspective, volunteer-led conversations can be interpreted as creating temporal relief within the institutional setting (see Results, section “A shift in perspective and distraction from ward routine”). Rather than adding content, such encounters may “make time” in an environment otherwise shaped by acceleration, interruptions and task-oriented routines. Relational presence thus acquires ethical significance not only through what is said but through the provision of unhurried time as a scarce care resource in inpatient palliative care [17].

Of particular relevance is the observation that care was experienced as meaningful even beyond verbal communication. Non-verbal presence, quiet companionship or physical closeness were described by volunteers as calming and supportive for patients (see Results, section “Human presence as a core element of support”). This is consistent with studies highlighting non-verbal responsiveness as an expression of dignity and recognition, particularly in phases of high symptom burden [15, 18].

Within this relational practice, two closely related forms of psychosocial support can be distinguished: attentive listening characterised by full presence, and more actively facilitated psychosocial conversations (see Results, sections “Relief through attentive listening with full presence” and “Psychosocially supportive conversations beyond family and staff”). While these modes are often subsumed under a single category of emotional support in the literature, the present findings suggest that they involve different demands on volunteers’ attitudes and competencies. Comparable distinctions have been described in international studies on hospice and palliative care volunteering [19, 20].

The relational orientation of the offer is closely linked to its structural characteristics, particularly its low-threshold and non-imposing nature (see Results, sections “Voluntariness and respect for refusal” and “Non-binding encounters with unfamiliar volunteers”). From volunteers’ perspectives, low-threshold access, non-binding encounters and the rotation of companions appear to facilitate patient openness, particularly regarding sensitive topics patients may wish to withhold from relatives (see Results, section “Non-binding encounters with unfamiliar volunteers”). This “outsider position”, conceptualised as a liminal space between family and clinical staff, functions as a relational resource [21, 22], enabling closeness without professional agenda and distance without familial entanglement [23]. In this sense, volunteers may also function as a link to the community within institutional palliative care, complementing clinical routines with relational and social resources and contributing not only at the bedside but also at the interface between institutions and social networks [5].

At the same time, the rotating format was experienced as emotionally demanding due to repeated first encounters and limited insight into subsequent patient trajectories (see Results, sections “Emotional demands of serial first encounters with unfamiliar patients” and “Lack of information about subsequent patient trajectories”). However, it may also be understood as an ethically protective boundary mechanism that helps prevent over-attachment and role drift that can arise in sustained one-to-one accompaniment [24]. This supports interpreting rotating conversation offers as a distinct form of accompaniment with specific mechanisms rather than as a deficit model [4].

Positive experiences were closely tied to the specialised palliative care setting. Interdisciplinary collaboration, a culture of appreciation and effective physical symptom control were described as essential prerequisites for psychosocial conversations (see Results, section “Protected environment and good symptom control as prerequisites and Good physical symptom control as a precondition for conversations”). Existing studies suggest that stable medical and nursing care may constitute a necessary condition for patients to engage with psychosocial concerns [4].

Against this background, low-threshold volunteer encounters can also be interpreted at an organisational level as intentional interruptions of clinical momentum. Such micro-pauses may counterbalance procedural density and support attentiveness and reflection, thereby contributing to person-centred care without competing with clinical priorities. From this perspective, the value of the offer lies not in replacing professional care but in modulating the rhythm of care delivery [25].

Interprofessional collaboration emerged as a further enabling factor. Information exchange between nursing staff and volunteer hospice companions facilitated situationally appropriate conversations but required clear role definitions, transparent communication and careful handling of sensitive information (see Results, section “Informational exchange with nursing staff”) [26, 27]. Clear role definitions appear particularly important because volunteers continuously negotiate boundaries between being “part of the team” and remaining distinct from professional care (see Results, section “Sense of belonging to the ward team”). Boundary ambiguity may increase emotional load and create ethical risks related to expectations, responsibility and information handling [24].

At the same time, the volunteer role was associated with emotional demands. Repeated first encounters, the young age of some patients and the absence of feedback regarding patient outcomes were described as particularly challenging (see Results, sections “Emotional demands of serial first encounters with unfamiliar patients”–“Confrontation with younger critically ill patients”) [4, 20]. Regular supervision and peer support were consistently identified as key protective factors, in line with research on emotional labour in palliative care contexts (see Results, section “Supervision as part of psychological self-care”) [19, 20, 26]. From an ethical perspective, supervision and peer exchange can be understood as part of the institutional infrastructure of volunteer programmes, helping to sustain integrity and well-being under moral adversity and shifting responsibility from individual coping to shared organisational accountability for ethically safe practice [28].

Interpreting these challenges solely at the level of individual experience, however, risks overlooking their structural character. Limited insight into subsequent patient trajectories can be situated within broader discussions of uncertainty as an inherent feature of serious illness care. Rather than representing a remediable deficit, certain forms of not-knowing are structurally embedded and ethically relevant, requiring recognition, support and reflective handling rather than resolution [29].

The explanatory scope of the study is limited to the perspectives of volunteer hospice companions. Nevertheless, the findings provide valuable insights into processes, conditions and challenges associated with a relatively underexplored form of support in hospital-based palliative care. While transferability is limited to settings with comparable structural and cultural characteristics, core principles such as low-threshold access, open-endedness and clearly defined roles may remain relevant beyond the immediate study context. Existing literature on hospice and palliative care volunteering remains dominated by descriptive work, with limited evidence on how volunteer roles can be designed, trained and embedded in complex institutional settings, supporting the positioning of the present study as addressing a specific organisational and practice-based gap [30].

Taken together, the findings suggest that the conversation offer should not be understood merely as an additional psychosocial service but as a specific form of relational care. Volunteers described their practice as situationally responsive, deliberately non-goal-oriented and closely aligned with patients’ willingness to engage. Care was enacted primarily through attentiveness and relational restraint rather than through predefined interventions or problem-solving strategies, resonating with ethical frameworks that conceptualise care as a relational practice whose normative quality derives from the appropriateness of relationship formation [31].

Within this framework, the findings can also be interpreted in relation to the tension between care and autonomy. Relational conceptions of autonomy emphasise that self-determination in serious illness depends on supportive social relationships rather than being exercised independently of them [8, 32]. The practice described in this study, avoiding coercion, respecting refusal and allowing silence, can therefore be understood as an ethically relevant form of autonomy support. In this sense, autonomy is not maximised but protected by preventing overload and implicit social pressure, consistent with relational accounts that emphasise reducing burdensome demands rather than maximising choice [8].

The findings further illustrate that care in palliative settings is necessarily normatively structured. Volunteer hospice companions continuously navigate boundaries between closeness and distance, initiative and restraint. Such boundary decisions cannot be fully secured through formal role definitions alone but require situational ethical judgement. Accordingly, medical ethics literature characterises care as a morally demanding practice marked by context sensitivity and tolerance of ambiguity [33], and the present findings offer an empirical illustration of contemporary ethical analyses of relational care in palliative contexts [34].

Moreover, the results suggest that relational care in palliative settings extends beyond patients alone. Although relatives were not explicitly studied, volunteers’ accounts repeatedly referred to their indirect involvement. The palliative care literature increasingly recognises relatives not merely as functional contributors to patient care but as moral agents with distinct support needs [35]. Low-threshold conversation offers may therefore contribute to stabilising relational constellations even when primarily patient-focused.

Finally, the emotional burdens described by volunteers highlight that care is always delivered under conditions of limitation. This included the fixed weekly time window and the need to coordinate visits with ward routines, which could make encounters feel intrusive at inconvenient moments (see Results, section “Limited temporal flexibility and coordination with ward routines”). Time constraints, incomplete information and emotional strain represent not only organisational but also ethical challenges. Care-ethical approaches emphasise that moral obligations are not unlimited and require transparent and fairly distributed support structures [36]. Supervision, clear role clarification and institutional embedding thus represent not merely practical prerequisites but expressions of shared moral responsibility.

Conceptually, the low-threshold design can therefore be understood not only as a pragmatic access strategy but as an institutional response to the moral challenges inherent in palliative care work. When care is delivered under time pressure and incomplete information, relational support becomes ethically charged and requires organisational structures that explicitly acknowledge moral complexity rather than placing the burden on individuals alone [36].

In summary, this study supports an understanding of volunteer-led conversation offers on hospital palliative care units as a distinct, relationally grounded form of care. Such offers may contribute to preserving autonomy, dignity and relationality under conditions of high vulnerability without replacing professional care, thereby extending the discourse on institutional palliative care through an empirically informed care-ethical perspective.

## Limitations

This study reflects only the perspectives of volunteer hospice companions and does not include patient-, relative- or professional staff–reported experiences, which limits conclusions regarding patient-centred outcomes [11]. This focus is nonetheless appropriate for an initial exploration of a low-threshold intervention that is primarily enacted and shaped by volunteers themselves.

All participants were recruited from a single hospice organisation and two palliative care units, which may restrict transferability; at the same time, the shared training background and established collaboration with the ward enhanced the internal coherence of the findings [37].

The qualitative design and reliance on self-reported data preclude conclusions about effectiveness or causality [38] and entail potential risks of recall or social desirability bias [39]. Methodological rigour was strengthened through independent double coding, consensus-based analysis [40], and interviews conducted by a researcher without a prior relationship to participants [11].

Finally, the rotating model limited insight into longer-term relational processes; however, as rotation was integral to the intervention’s low-threshold design, this aspect can be understood analytically as a characteristic of the intervention rather than solely as a limitation [41].

## Conclusion

This qualitative interview study suggests that low-threshold, non-prearranged conversations provided by trained volunteer hospice companions constitute a distinct form of relational care within hospital palliative care. The offer is characterised by open-endedness, situational responsiveness and non-imposition, including respect for silence and refusal, rather than by predefined goals or outcomes.

For practice, the findings indicate that such conversation offers can be sustainably integrated into inpatient palliative care when embedded in a supportive institutional framework. Key enabling conditions include clearly defined volunteer roles, structured and consent-based communication with the interprofessional team, and regular supervision to address emotional demands. Training programmes should explicitly prepare volunteers for both attentive listening and more actively facilitated psychosocial conversations.

Future research should incorporate patient and family perspectives and compare rotating and continuity-based volunteer models across different inpatient contexts, including outcomes relevant to relational care, autonomy and psychosocial well-being.

## Supplementary Information

Below is the link to the electronic supplementary material.

Supplementary Material 1

## Data Availability

The audio-taped and transcribed interviews are not publicly available due to private details about the participants.

## Abbreviations

COREQ: Consolidated Criteria for Reporting Qualitative Research.
