# Supplementary material for: Relational care in palliative care units: a qualitative study of low-threshold volunteer hospice conversations
Source: BMC Health Serv Res. 2026 Apr 11;26:527. doi: 10.1186/s12913-026-14399-0 (PMC13085311; doi:10.1186/s12913-026-14399-0)
Supplement: Supplementary file 1 — Supplementary Material 1 [file 12913_2026_14399_MOESM1_ESM.docx]

**Appendix 1. Semi-structured interview guide**

Experience of the conversation offer

1. How does a typical visit to the palliative care unit usually unfold for you?
2. What do you see as the purpose and value of this low-threshold conversation offer on the palliative care unit?
   - Perceived effectiveness
   - Contribution compared with other forms of support

Content and relational aspects

1. Which topics typically arise in conversations with patients?
   - Experiences of talking about issues patients may not share with others
2. How would you describe your role as a volunteer in this setting?
   - Nature and quality of relationships in this format
   - Differences compared with continuity-based hospice accompaniment

Setting-specific factors

1. How does the inpatient palliative care setting shape your work?
   - Differences from home or hospice settings
   - Influence of symptom control, ward routines, and team structure

Challenges and burden

1. What challenges or limits do you experience in this format?
   - Emotional demands
   - Situations that feel particularly difficult
2. What kinds of support (e.g. training, supervision) are helpful or would be helpful for you in this context?

Involvement of relatives

1. Have you also had conversations with relatives?
   - Typical topics
   - Perceived effects of these conversations

Collaboration with the ward team

1. How would you describe collaboration with the palliative care team?
   - Role clarity
   - Information exchange
   - Suggestions for improvement

Closing

1. Is there anything else you consider important that we have not yet discussed?
